# Trends and future predictions of chronic kidney disease due to diabetes mellitus type 2 attributable to dietary risks: insights based on GBD 2021 data

**DOI:** 10.3389/fnut.2024.1494383

**Published:** 2025-01-13

**Authors:** Ming Xu, Hongxia Wei, Dongqing Lv, Yanhong Wei, Ziang Liu, Yi Zhang, Yunfeng Liu

**Affiliations:** ^1^Department of Endocrinology, First Hospital of Shanxi Medical University, Taiyuan, China; ^2^The First Clinical Medical College, Shanxi Medical University, Taiyuan, China; ^3^Department of Pharmacology, School of Basic Medicine, Shanxi Medical University, Taiyuan, China; ^4^Medicinal Basic Research Innovation Center of Chronic Kidney Disease, Ministry of Education, Shanxi Medical University, Taiyuan, China

**Keywords:** diabetes mellitus type 2, chronic kidney disease, dietary risk, GBD database, BapC, Bayesian age-period-cohort

## Abstract

**Background:**

The 2021 Global Burden of Disease (GBD) study shows a continuous increase in the burden of chronic kidney disease due to diabetes mellitus type 2 (CKD-T2DM) from 1990 to 2021. This study examines the influence of dietary risk factors across various populations and socioeconomic groups.

**Methods:**

Utilizing the 2021 GBD data, we analyzed age-standardized CKD-T2DM metrics—including mortality, disability-adjusted life years (DALY), and age-standardized rates (ASR)—stratified by age, gender, and region. The study employs estimated annual percentage changes (EAPC) to monitor temporal trends and project future trends from 2022 to 2035 using bayesian age-period-cohort (BAPC) analysis.

**Results:**

The results indicate that, in 2021, 20.55% of CKD-T2DM mortality and 23.21% of CKD-T2DM DALY were attributed to poor diets, especially those low in fruits and high in red and high processed meat. Throughout this period, both mortality and DALY rates associated with dietary risks increased significantly, with the most rapid increase in diet high in sugar-sweetened beverages, highlighting the considerable impact of dietary factors on the global CKD-T2DM landscape. Geographic disparities in T2DM trends are evident, with the most significant increases in age-standardized mortality rates (ASMR) and age-standardized DALY rates (ASDR) observed in regions such as high-income North America and Central Latin America. Socio-demographic index (SDI) is negatively correlated with the CKD-T2DM burden attributable to dietary risk factors.

**Conclusion:**

Public health interventions that target dietary changes can significantly reduce the global burden of CKD-T2DM.

## 1 Introduction

In recent decades, the global prevalence of type 2 diabetes mellitus (T2DM) has surged significantly, imposing not only a personal burden but also straining healthcare systems and economies worldwide (1). Approximately 40% of individuals with diabetes will develop diabetic nephropathy, a leading cause of chronic kidney disease (CKD) worldwide, with a continuously rising burden (2).

The role of dietary factors, such as excessive intake of red and processed meat, high consumption of sugar sweetened beverages (SSBs) and insufficient intake of fruits and vegetables, in the onset and progression of diabetic nephropathy has been extensively studied (3–8). Nephrologists recommend that CKD patients limit protein intake primarily to reduce the accumulation of these molecules and mitigate the occurrence and severity of uremic symptoms (3). High sodium intake raises blood pressure and proteinuria, serving as a risk factor for CKD progression, while moderate sodium restriction can reduce urinary albumin excretion (9–11). Overall, nutritional components play an essential role in the etiology and progression of CKD. They primarily contribute by disrupting lipid absorption and metabolism, inducing oxidative stress secondary to hyperglycemia, and propagating inflammatory processes (12). These pathophysiological mechanisms collectively lead to renal damage, manifested as the deterioration of both kidney structure and function (13).However, the specific burden of diabetic nephropathy attributable to dietary risks remains largely unexplored.

The Global Burden of Disease (GBD) study, initiated by the Institute for Health Metrics and Evaluation at the University of Washington, is a global collaborative research project aimed at assessing the impact of diseases, disabilities, and deaths on socioeconomic factors and health, including the study of disease risk factors. This assessment methodology facilitates the analysis and comparison of health data across different diseases, time periods, and regions, and is internationally recognized as a comprehensive system for disease burden evaluation (14). During the entire study period up until 2021, dietary risks remained one of the leading global risk factors. Among specific major dietary risk factors, such as low consumption of fruits, whole grains, and vegetables, and high sodium intake, the attributable DALY count continues to rise. Furthermore, these dietary risks indirectly contribute to various metabolic risk factors, such as elevated fasting blood glucose (FGP) and high systolic blood pressure (SBP), which are key contributors to the global disease burden (15).

Therefore, this study aims to provide a detailed description of the global landscape of CKD-T2DM attributable to seven dietary risk factors, contributing to a deeper understanding of the role of dietary risks in mortality and disability across various populations and socioeconomic groups, while elucidating trends over the past 32 (1990–2021) years. By comprehensively analyzing the impact of dietary risks on CKD-T2DM, this research seeks to fill existing gaps in the literature, incorporating data from 21 GBD regions, 5 SDI regions, and 204 countries and territories regarding seven dietary risk factors. Through enhancing the understanding of the global burden of CKD-T2DM and its dietary determinants, we aim to contribute to international efforts to mitigate the impact of this chronic disease on public health.

## 2 Materials and methods

### 2.1 Data sources and definitions

This epidemiological study utilized retrospective data extracted from the GBD 2021 database, managed by the Institute for Health Metrics and Evaluation (IHME).^1^ The GBD 2021 study encompasses comprehensive demographic and epidemiological information from 204 countries and territories between 1990 and 2021. This data is sourced from various inputs, including censuses, household surveys, and disease registries. The current study incorporates annual age-adjusted information on the burden of CKD-T2DM attributable to dietary risk factors from 1990 to 2021, stratified by age, sex, and region. The data spans various populations across 204 countries/territories, 21 GBD regions, and 5 socio-demographic index (SDI) regions.

### 2.2 Estimation of the burden of CKD-T2DM

The metrics of interest include the age-standardized mortality rate (ASMR) and age-standardized disability-adjusted life year (ASDR) rate per 100,000 individuals, as well as mortality and DALY by age group. The International Classification of Diseases, 10th Edition (ICD-10) was used as the coding guideline for CKD-T2DM. Comprehensive information on data sources and related metadata is available through the online data platform at: http://ghdx.healthdata.org/gbd-2021/data-input-sources (accessed August 25, 2024).

### 2.3 Estimation of attributable burden

The proportion of CKD-T2DM burden attributable to dietary risk factors was calculated using Population Attributable Fraction (PAF) (14). PAF quantifies the extent of impact, representing the fraction of disease outcomes that could potentially be avoided if the risk factor were eliminated from the population. It is estimated by comparing the theoretical minimum risk exposure level with the actual population exposure level, assuming all other risk factors remain constant. The attributable burden is derived by multiplying the relevant PAF by the total CKD-T2DM burden for each age, sex, location, and year group. The calculation of PAF is complex, and in 2021, it was further refined with the addition of the Burden of Proof Risk Function (BPRF), which provides a more conservative estimate of the risk-outcome relationship. This method also accounts for heterogeneity across different studies and settings, improving the robustness of the risk assessment (16, 17).

### 2.4 Selection of dietary risk factors

For CKD-T2DM, data on seven specific dietary risk factors was collected from the Global Health Data Exchange (GHDX) query tool: http://ghdx.healthdata.org/gbd-results-tool (accessed August 25, 2024). These risk factors include low intake of fruits, vegetables, and whole grains, as well as high intake of red meat, high processed meats, sodium, and SSBs. All dietary risk factors are based on a 24-h dietary recall survey, with food and nutrient consumption reported in grams per person per day.

### 2.5 Socio-demographic index

The GBD 2021 study uses the Socio-Demographic Index (SDI) as a composite measure of socio-economic status closely related to health outcomes. SDI combines per capita income, educational attainment, and total fertility rate (TFR) to assess the socio-demographic development of a country. According to the GBD 2021 study, SDI is categorized into five levels: high SDI (>0.81), high-middle SDI (0.70–0.81), middle SDI (0.61–0.69), low-middle SDI (0.46–0.60), and low SDI (<0.46).

### 2.6 Statistical analysis

To assess the burden of CDK-T2DM attributable to dietary risk factors, variables such as mortality numbers, DALY, and their age-standardized rates (ASR) within 95% uncertainty intervals (UI) were used. To measure temporal changes in age-standardized mortality rates and DALY rates attributable to dietary factors from 1990 to 2021, the estimated annual percentage change (EAPC) was employed (18). EAPC was calculated using a regression model that describes the pattern of age-standardized rates over a specific period. The equation used: Y = *α* + *β*X + e, where Y represents the natural logarithm of ASR, X represents calendar year, α is the intercept, β is the slope or trend, and e is the error term. EAPC is computed as 100 × [exp(β) – 1], reflecting the annual percentage change. Linear regression models were used to calculate the 95% confidence interval (CI) for EAPC, and spearman correlation analysis was conducted to explore the relationship between ASRs and SDI values. Bayesian age-period-cohort (BAPC) analysis for ASR was performed using the BAPC and Integrated Nested Laplace Approximation (INLA) packages to predict trends attributable to dietary factors in CDK-T2DM. The model employed a Poisson framework and categorized the data by country, gender, and age, fitting it within the INLA approach. Age, period and/or cohort effects were modeled using either a second-order random walk (RW2) or fixed effect (drift). The BAPC model effectively circumvents the complexities associated with Markov Chain Monte Carlo (MCMC) sampling, making it more practical for real-world application. Additionally, it delivers well-calibrated probabilistic forecasts with narrow prediction intervals, outperforming the generalized Lee-Carter model (19). All statistical analyses were conducted using RStudio (version 4.3.0), with data visualization created using the ggplot2 package.

## 3 Results

### 3.1 Global trends in the burden of CDK-T2DM attributable to dietary risk factors from 1990 to 2021

Analysis of the GBD 2021 data reveals that in 2021, 16.79% of CDK-T2DM deaths (95% UI: 7.62, 26.33) and 17.76% of CDK-T2DM DALYs (95% UI: 27.60, 8.08) were attributable to dietary risk factors. This represents a decrease from 18.45% (95% UI: 7.91, 28.83) and 19.41% (95% UI: 8.16, 30.32) in 1990, respectively. However, the total number of CDK-T2DM deaths attributable to dietary risks increased significantly from 27,231.78 (95% UI: 42,888.58, 11,100.99) in 1990 to 79,988.20 (95% UI: 128,88,32,73) in 2021, while the number of DALYs also rose from 798,300 (95% UI: 127,74,32.28) to 1,999,200 (95% UI: 316,72,85.62) over the same period (Supplementary Table 1).

**Figure 1:**
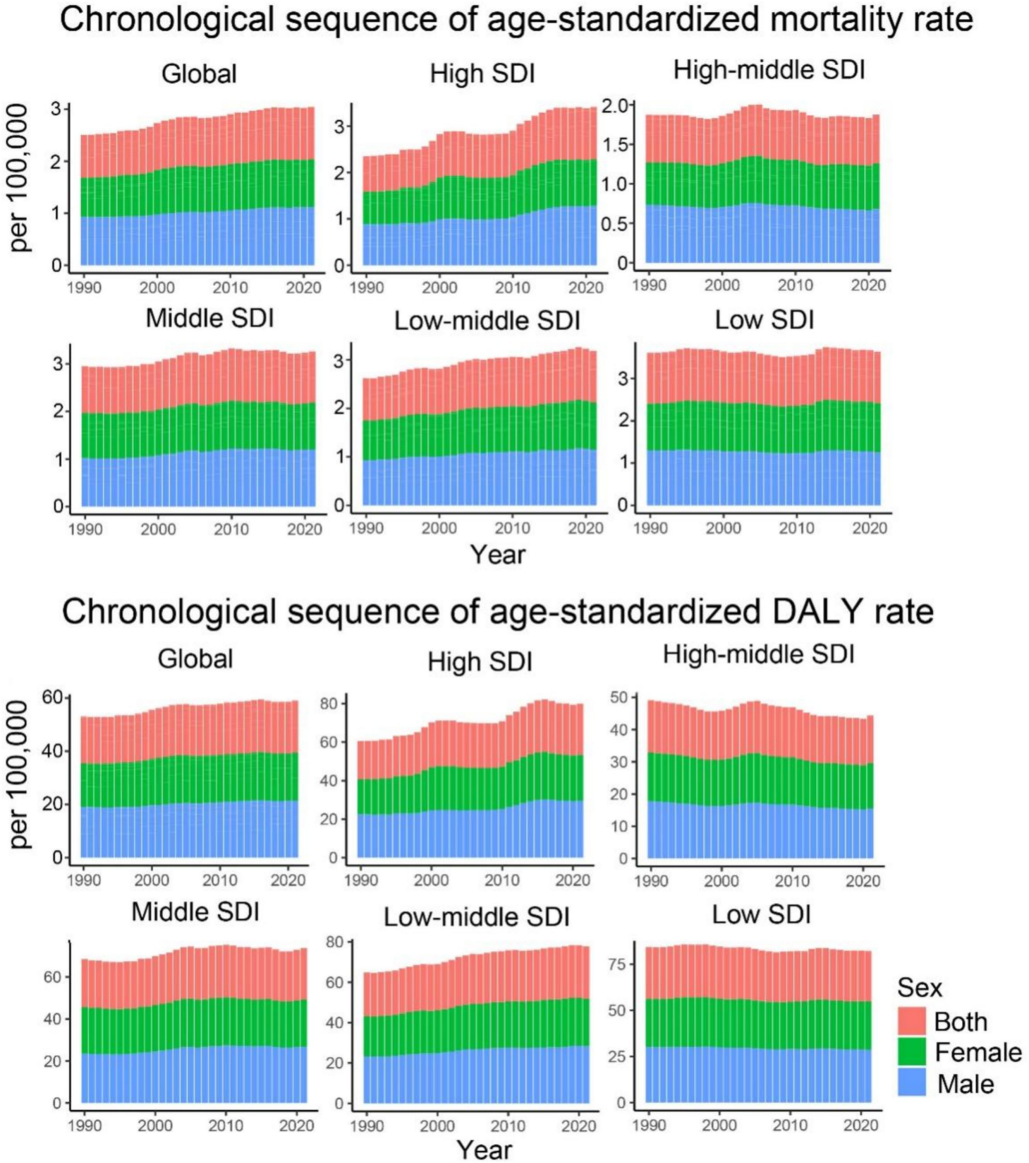
Age-standardized mortality and DALY rates for CKD-T2DM attributable to dietary risk factors from 1990 to 2021 by SDI regions and sex.

In 2021, the age-standardized mortality rates (ASMRs) and age-standardized disability rates (ASDRs) for CDK-T2DM attributable to dietary risks were 0.958 per 100,000 (95% UI: 0.395, 1.539) and 23.206 per 100,000 (95% UI: 9.953, 36.613), respectively. From 1990 to 2021, ASMRs (EAPC: 0.76; 95% CI: 0.69, 0.83) and ASDRs (EAPC: 0.47; 95% CI: 0.41, 0.53) attributable to dietary risk factors showed significant increasing trends (Table 1, Figure 1). Our analysis identified the three dietary risk factors with the greatest contribution to ASMR and ASDR for CDK-T2DM from 1990 to 2021: diet low in fruits, diet low in whole grains, and diet high in processed meat (Table 1).

**Table 1:** Global age-standardized rates and rates changes attributable to seven dietary factors for CKD-T2DM burden, 1990 and 2021.

Dietary factor	Age-standardized rate per 100, 000 People (95% UI)				Estimated annual percentage change from 1990 to 2021 (95% CI)	
	1990		2021			
	Death rate	DALY rate	Death rate	DALY rate	Death rate	DALY rate
Dietary risks	0.776 (0.315, 1.234)	20.550 (8.424, 32.261)	0.958 (0.395, 1.539)	23.206 (9.953, 36.613)	0.76 (0.69, 0.83)	0.47 (0.41, 0.53)
Diet low in fruits	0.228 (0.079, 0.405)	6.019 (2.230, 10.523)	0.246 (0.087, 0.452)	5.980 (2.218, 10.705)	0.22 (0.17, 0.27)	−0.05 (−0.10, −0.00)
Diet low in whole grains	0.182 (0.049, 0.321)	4.762 (1.255, 8.589)	0.200 (0.052, 0.358)	4.864 (1.250, 8.806)	0.33 (0.27, 0.40)	0.10 (0.03, 0.17)
Diet high in processed meat	0.132 (0.037, 0.229)	3.885 (1.006, 6.886)	0.176 (0.046, 0.296)	4.385 (1.109, 7.643)	1.09 (0.95, 1.23)	0.55 (0.42, 0.68)
Diet high in red meat	0.122 (0.000, 0.269)	3.336 (0.000, 7.012)	0.169 (0.000, 0.368)	4.183 (0.000, 8.992)	1.23 (1.13, 1.32)	0.91 (0.82, 1.00)
Diet low in vegetables	0.116 (0.028, 0.245)	2.966 (0.729, 6.104)	0.132 (0.033, 0.296)	3.123 (0.826, 6.880)	0.53 (0.49, 0.57)	0.23 (0.20, 0.27)
Diet high in sodium	0.061 (0.004, 0.217)	1.499 (0.098, 5.445)	0.074 (0.001, 0.298)	1.703 (0.035, 6.887)	0.53 (0.47, 0.58)	0.32 (0.27, 0.37)
Diet high in sugar-sweetened beverages	0.032 (0.017, 0.052)	0.910 (0.469, 1.417)	0.059 (0.031, 0.088)	1.550 (0.801, 2.353)	2.07 (1.91, 2.22)	1.91 (1.79, 2.03)

CI, confidence interval; DALY, disability-adjusted life year; UI, uncertainty interval.

Geographically, there is considerable heterogeneity in the trends of CDK-T2DM attributable to dietary risks. The highest annual increase in ASMR was observed in High-income North America (+3.18%) and Central Latin America (+2.03%), followed by Australasia (+1.68%) and the Caribbean (+1.37%). A similar regional trend was seen for ASDR. Conversely, the regions with the largest annual decrease in ASMR were High-income Asia Pacific (−1.54%), Central Sub-Saharan Africa (−0.76%), and East Asia (−0.57%). The greatest annual decrease in ASDR occurred in High-income Asia Pacific (−1.37%), Eastern Europe (−1.11%), and Central Sub-Saharan Africa (−0.80%) (Table 2).

**Table 2:** Age-standardized rates (1990 and 2021) and estimated annual percentage change (EAPC, from 1990 to 2021) for CKD-T2DM attributable to dietary factors by SDI and regions (Bolded regions are GBD super-regions).

Location	Age-standardized rate per 100, 000 people (95% UI)				Estimated annual percentage change from 1990 to 2021 (95% CI)	
	1990		2021			
	Death Rate	DALY rate	Death Rate	DALY rate	Death rate	DALY rate
Global risks	0.776 (0.315–1.234)	20.550 (8.424–32.261)	0.958 (0.395–1.539)	23.206 (9.953–36.613)	0.76 (0.69, 0.83)	0.47 (0.41, 0.53)
**Central Europe, Eastern Europe, and Central Asia**	0.232 (0.112–0.350)	11.783 (5.785–17.372)	0.286 (0.141–0.452)	10.429 (5.341–15.559)	0.49 (0.36, 0.62)	−0.63 (−0.72, −0.54)
Central Asia	0.224 (0.114–0.349)	15.569 (7.855–23.689)	0.408 (0.197–0.643)	16.798 (8.368–25.054)	1.34 (0.86, 1.82)	−0.09 (−0.34, 0.17)
Central Europe	0.346 (0.165–0.543)	11.994 (6.099–17.997)	0.289 (0.133–0.474)	9.618 (4.945–15.098)	−0.55 (−0.80, −0.30)	−0.58 (−0.70, −0.45)
Eastern Europe	0.172 (0.074–0.261)	10.990 (5.085–16.426)	0.248 (0.117–0.387)	9.004 (4.484–13.601)	0.81 (0.37, 1.26)	−1.11 (−1.28, −0.94)
**High-income**	0.701 (0.320–1.075)	19.649 (9.145–29.920)	1.008 (0.434–1.541)	25.212 (10.966–38.256)	1.32 (1.19, 1.45)	0.96 (0.84, 1.08)
Australasia	0.180 (0.081–0.287)	7.472 (3.439–11.522)	0.240 (0.098–0.406)	8.372 (3.700–13.025)	1.68 (1.22, 2.14)	0.60 (0.35, 0.84)
High-income North America	0.736 (0.305–1.153)	23.000 (9.348–35.738)	1.897 (0.714–2.908)	47.821 (17.943–73.134)	3.18 (2.91, 3.46)	2.53 (2.30, 2.77)
High-income Asia Pacific	1.341 (0.681–1.995)	29.201 (15.282–43.023)	0.856 (0.370–1.313)	18.929 (8.981–28.321)	−1.54 (−1.65, -1.43)	−1.37 (−1.51, -1.23)
Southern Latin America	1.443 (0.518–2.470)	32.570 (12.211–53.522)	1.106 (0.444–1.836)	24.258 (9.928–39.122)	−0.47 (−0.85, −0.09)	−0.61 (−0.92, −0.30)
Western Europe	0.462 (0.205–0.745)	14.039 (6.414–21.637)	0.460 (0.205–0.747)	11.893 (5.348–18.392)	0.46 (0.22, 0.69)	−0.37 (−0.47, −0.26)
**Latin America and Caribbean**	1.308 (0.522–2.159)	31.836 (13.139–51.439)	1.712 (0.721–2.834)	41.169 (18.045–66.897)	1.20 (0.90, 1.49)	1.07 (0.78, 1.36)
Caribbean	1.323 (0.523–2.198)	31.484 (12.925–50.635)	1.697 (0.740–2.949)	39.956 (18.016–66.792)	1.37 (1.17, 1.57)	1.28 (1.11, 1.45)
Andean Latin America	1.551 (0.510–2.767)	33.576 (11.370–58.712)	2.007 (0.728–3.634)	43.694 (16.771–76.516)	0.95 (0.76, 1.14)	0.95 (0.76, 1.15)
Central Latin America	1.165 (0.494–1.991)	28.827 (12.588–46.014)	1.790 (0.765–2.974)	44.938 (20.140–71.406)	2.03 (1.51, 2.55)	1.97 (1.48, 2.45)
Tropical Latin America	1.383 (0.574–2.254)	34.392 (14.813–54.832)	1.574 (0.662–2.522)	37.192 (16.044–59.240)	0.40 (0.17, 0.63)	0.15 (−0.09, 0.38)
**North Africa and Middle East**	0.859 (0.323–1.536)	20.372 (7.775–35.353)	0.735 (0.277–1.241)	17.650 (6.782–29.902)	−0.53 (−0.62, −0.44)	−0.48 (−0.52, −0.43)
**South Asia**	0.787 (0.322–1.340)	21.251 (8.664–34.935)	0.957 (0.375–1.682)	25.334 (10.525–43.100)	0.62 (0.56, 0.68)	0.61 (0.56, 0.65)
**Southeast Asia, East Asia, and Oceania**	0.895 (0.265–1.560)	20.819 (5.974–35.964)	0.838 (0.293–1.445)	19.232 (6.773–33.703)	−0.24 (−0.34, −0.13)	−0.19 (−0.31, −0.08)
Southeast Asia	0.786 (0.249–1.477)	19.276 (6.134–34.361)	0.899 (0.266–1.736)	21.506 (6.852–39.701)	0.53 (0.48, 0.58)	0.47 (0.41, 0.52)
East Asia	0.953 (0.301–1.672)	21.505 (6.326–37.832)	0.815 (0.264–1.434)	18.455 (5.748–32.680)	−0.57 (−0.70, −0.44)	−0.45 (−0.59, −0.31)
Oceania	1.129 (0.321–2.102)	28.154 (7.591–51.392)	1.378 (0.432–2.504)	31.787 (9.988–57.158)	0.56 (0.47, 0.66)	0.33 (0.25, 0.41)
**Sub-Saharan Africa**	1.173 (0.468–1.971)	28.360 (10.933–46.813)	1.243 (0.482–2.139)	28.693 (11.801–48.352)	0.11 (0.06, 0.16)	−0.03 (−0.07, 0.01)
Central Sub-Saharan Africa	1.592 (0.553–2.762)	38.499 (14.002–65.955)	1.369 (0.449–2.543)	32.624 (11.146–58.228)	−0.76 (−0.87, −0.66)	−0.80 (−0.89, −0.70)
Eastern Sub-Saharan Africa	1.813 (0.701–3.145)	39.335 (15.906–69.140)	1.994 (0.810–3.369)	40.985 (17.425–69.446)	0.18 (0.13, 0.23)	0.00 (−0.05, 0.05)
Southern Sub-Saharan Africa	0.519 (0.195–0.928)	16.667 (6.451–29.408)	0.672 (0.263–1.191)	19.565 (8.210–33.402)	1.27 (0.95, 1.59)	0.78 (0.54, 1.02)
Western Sub-Saharan Africa	0.796 (0.257–1.408)	20.690 (6.797–35.959)	0.767 (0.265–1.326)	20.178 (7.410–33.812)	−0.21 (−0.30, −0.13)	0.10 (−0.16, −0.04)
High SDI	0.702 (0.322–1.065)	19.924 (9.205–30.270)	1.058 (0.454–1.612)	26.483 (11.500–40.293)	1.45 (1.32, 1.58)	1.05 (0.93, 1.17)
High-middle SDI	0.567 (0.217–0.932)	16.113 (6.391–25.555)	0.589 (0.237–0.968)	14.698 (5.962–23.772)	0.11 (0.01, 0.21)	−0.30 (−0.39, −0.21)
Middle SDI	0.940 (0.346–1.589)	22.809 (8.399–37.404)	1.035 (0.397–1.728)	24.459 (9.869–39.174)	0.42 (0.33, 0.50)	0.34 (0.24, 0.44)
Low-middle SDI	0.835 (0.323–1.400)	21.655 (8.390–35.067)	1.012 (0.412–1.716)	25.884 (10.808–43.475)	0.68 (0.63, 0.73)	0.66 (0.60, 0.72)
Low SDI	1.150 (0.461–1.925)	28.192 (11.417–46.197)	1.157 (0.445–2.023)	27.408 (11.319–46.782)	0.00 (−0.07, 0.07)	−0.13 (−0.17, −0.09)

Regarding the trends of ASMR and ASDR for CDK-T2DM due to dietary risk factors across five different SDI regions, most regions (low, middle, and high SDI) exhibited a consistent upward trend. High SDI regions showed the greatest annual increase (EAPC of 1.45% for ASMR and 1.05% for ASDR), whereas middle SDI regions had annual growth rates of approximately 0.68% for ASMR and 0.66% for ASDR. In contrast, middle-high and low SDI regions showed markedly different trends, with a relatively stagnant annual increase in ASMR of 0.11 and 0.00%, respectively, and a decline in ASDR at rates of 0.30 and 0.13%, respectively. Despite fluctuations over the years, the overall growth in ASMR for middle SDI regions was approximately 0.42%, and the growth in ASDR was 0.34% (Figure 1).

**Figure 2:**
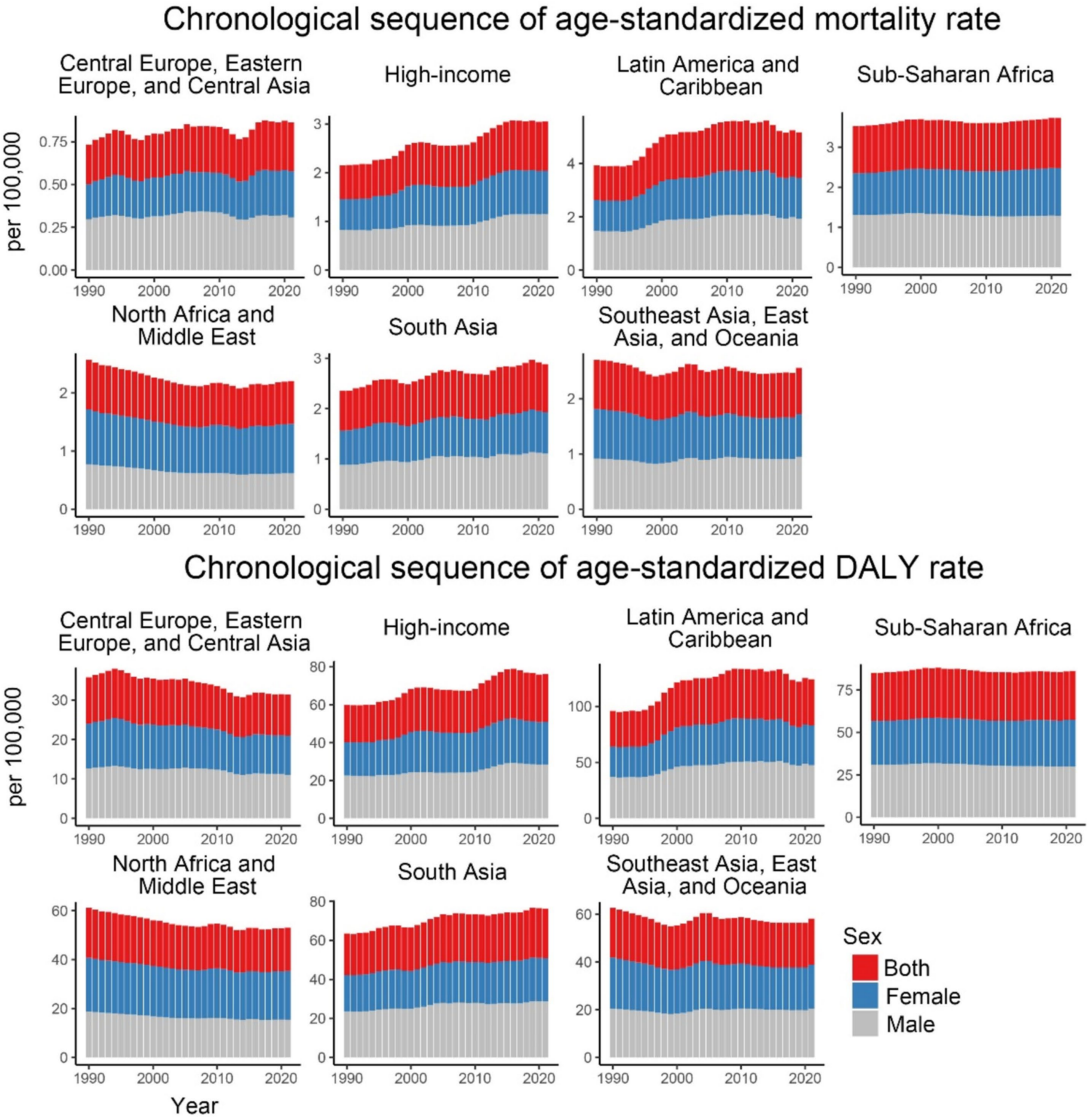
Age-standardized mortality and DALY rates for CKD-T2DM attributable to dietary risk factors from 1990 to 2021 by 7 GBD super regions and sex.

Over the past 32 years, notable trends have been observed in the ASMR across high-income regions, South Asia, Latin America, and the Caribbean. High-income regions showed the greatest fluctuations, while South Asia, Latin America, and the Caribbean experienced more moderate increases. Regional trends in ASDR indicate the highest values in Latin America and the Caribbean, with the fastest growth in high-income regions and South Asia. In contrast, regions such as Sub-Saharan Africa, Central Europe, Eastern Europe, Central Asia, Southeast Asia, East Asia, and Oceania exhibited a decline followed by stabilization, with Sub-Saharan Africa showing a continuous stable trend (Figure 2).

**Figure 3:**
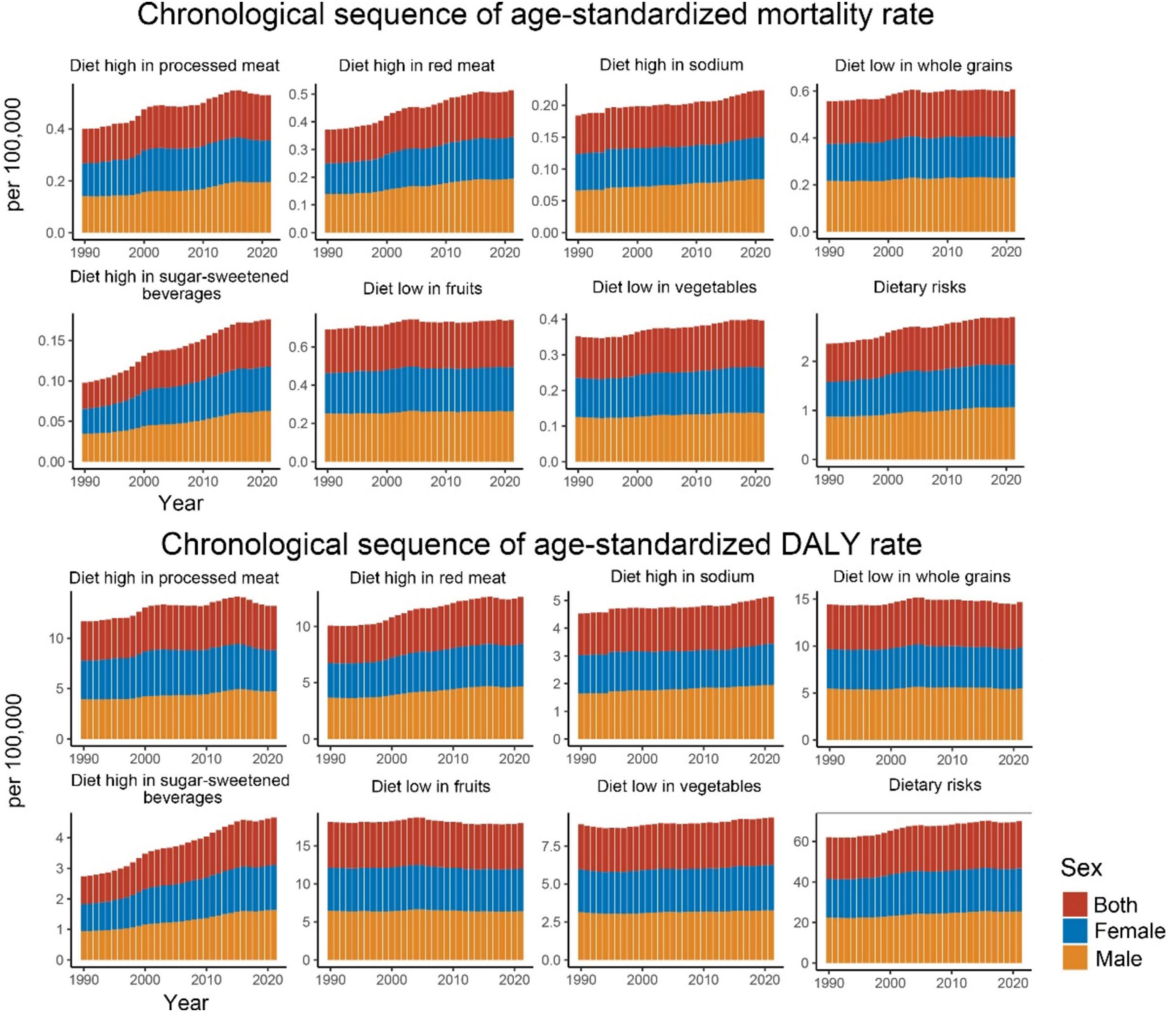
Age-standardized mortality and DALY rates for CKD-T2DM attributable to specific dietary risk factors from 1990 to 2021 by sex.

Regarding specific dietary factors (Figure 3 and Table 1), nearly all dietary risk factors for CDK-T2DM exhibited an increasing trend in ASMR and ASDR over the 32 years. The highest ASMR dietary risk factor was a diet low in fruits, with a rate of 0.246 per 100,000 people in 2021 (EAPC: 0.22, 95% UI: 0.17, 0.27). Significant annual increases were noted for diets high in SSBs (EAPC: 2.07, 95% UI: 1.91, 2.22), high in red meat (EAPC: 1.23, 95% UI: 1.13, 1.32), high in processed meat (EAPC: 1.09, 95% UI: 0.95, 1.23), and low in whole grains (EAPC: 0.33, 95% UI: 0.27, 0.40). For CDK-T2DM ASDR, the highest ASDR in 2021 was also attributed to a diet low in fruits, at 5.980 per 100,000 people, followed by a diet low in whole grains at 4.864 per 100,000 people. Among DALY rates attributable to dietary factors, the highest annual growth rates were observed for diets high in SSBs (EAPC: 1.91, 95% UI: 1.79, 2.03), high in red meat (EAPC: 0.91, 95% UI: 0.82, 1.00), and high in processed meat (EAPC: 0.55, 95% UI: 0.42, 0.68).

### 3.2 Global trends of dietary risk factors for CKD-T2DM by sex and age in 2021

**Figure 4:**
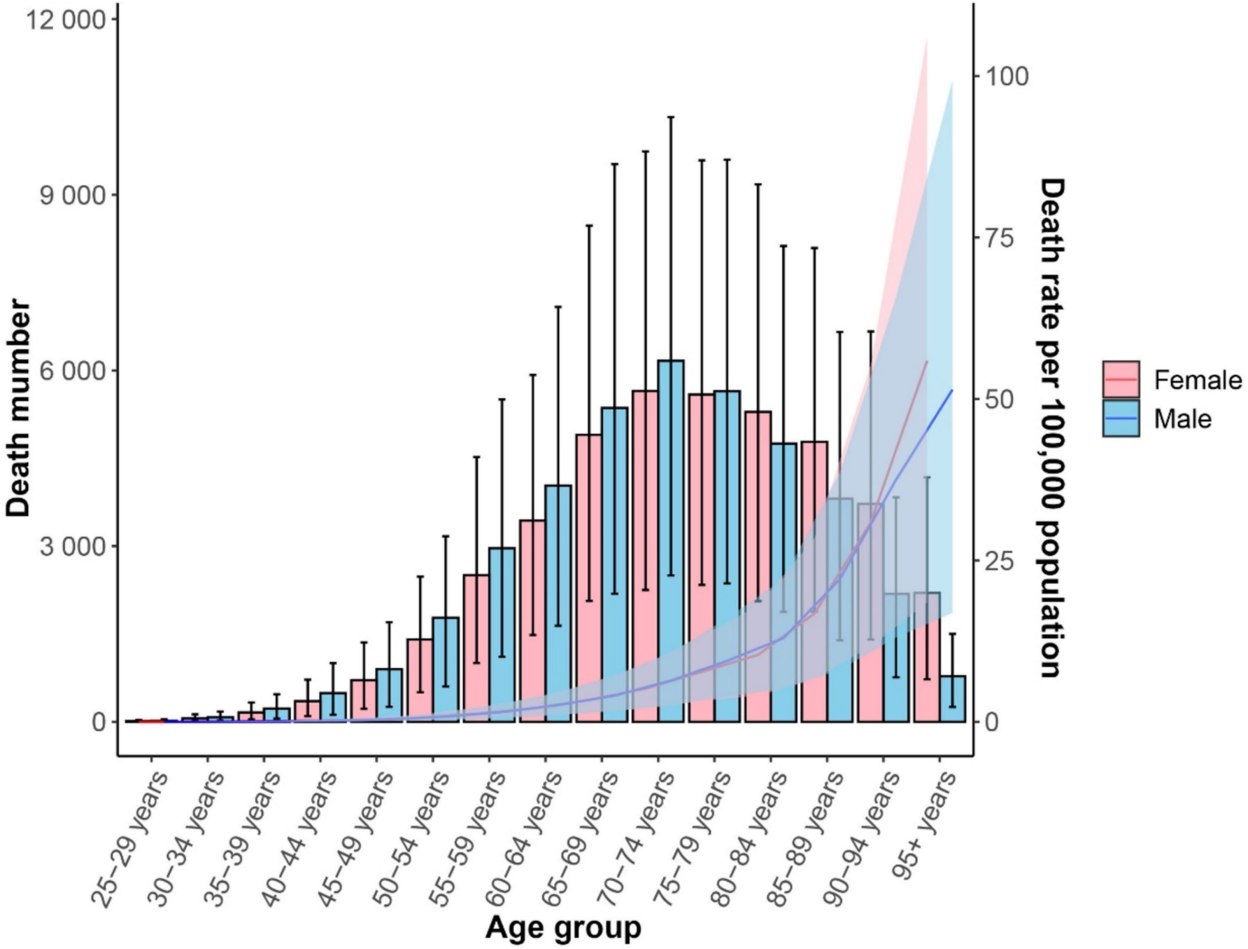
Age-standardized mortality rate (lines) and death number (bars) for CDK-T2DM attributable to dietary risk factors in 2021 by age and sex.

In 2021, the number of CKD-T2DM deaths attributable to dietary risk factors varied by age group, with the highest peaks observed in females and males aged 70–74. The mortality rate for CKD-T2DM attributable to dietary risk factors was higher in males compared to females. Furthermore, for individuals younger than 80–84 years, males had a higher number of CKD-T2DM deaths than females, but females had a higher number of deaths for those aged 80–84 years and older. Both male and female mortality rates attributable to dietary risk factors for CKD-T2DM increased with age, with males having higher rates than females in earlier age groups, and females having higher rates than males after age 90 (Figure 4). In 2021, the total number of deaths for females was 39,192 (95% UI = 16,130; 64,109), while for males it was 40,795 (95% UI = 16,960; 65,211).

**Figure 5:**
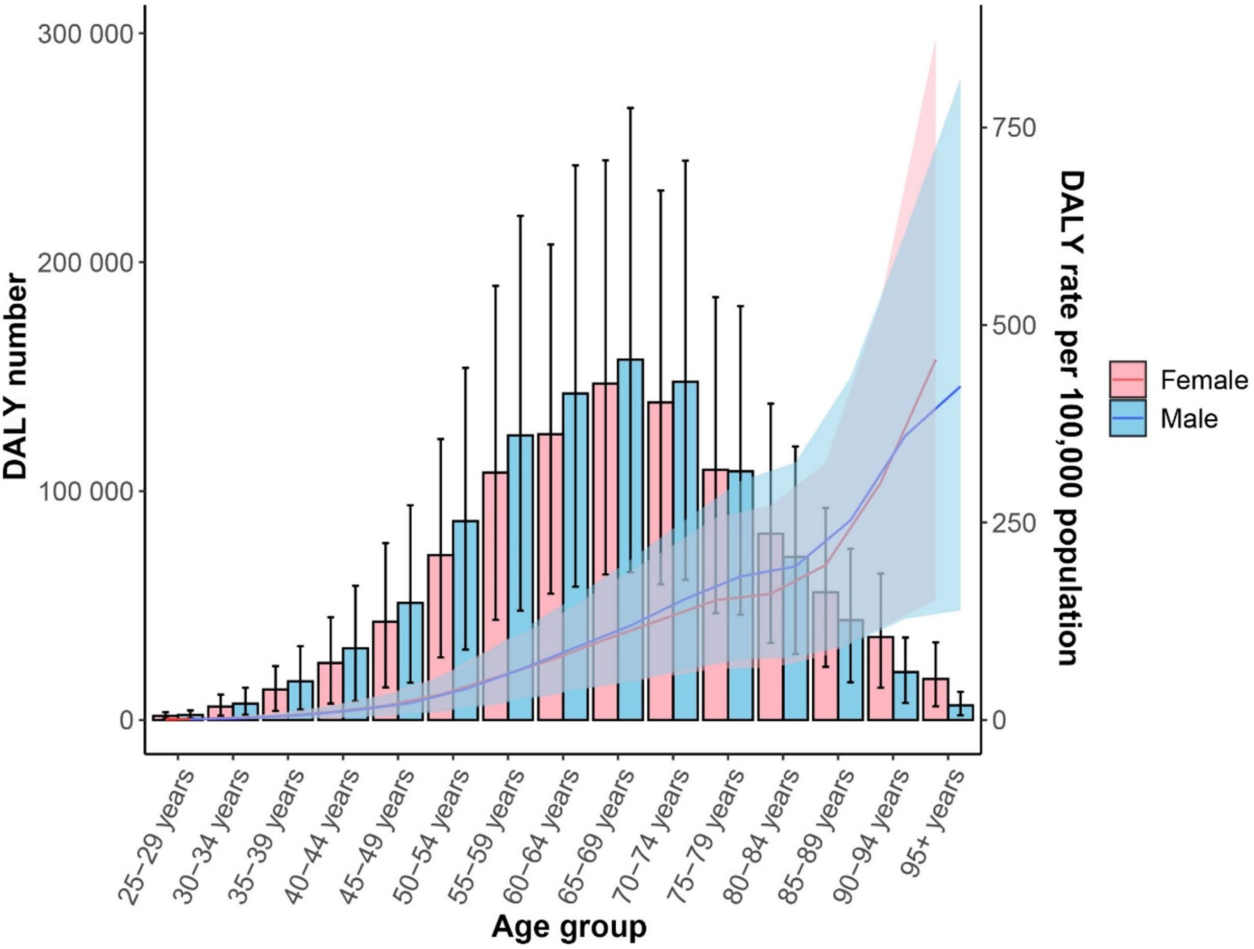
Age-standardized DALY rate per 100,000 people (lines) and DALYs number (bars) for CDK-T2DM attributable to dietary risk factors in 2021 by age and sex.

In 2021, the peak in DALYs attributable to dietary risk factors for CKD-T2DM was observed in males and females aged 65–69 years. Additionally, for individuals under 74 years, the DALYs due to dietary risk factors for CKD-T2DM were higher in males than in females, whereas for those aged 75–79 years and older, females had higher DALYs than males. The DALY rate for CKD-T2DM attributable to dietary risk factors was higher in males compared to females before around 90, and increased with age for both sexes (Figure 5). The total DALYs for females was 9.80 million (95% UI = 4.21; 15.46), and for males was 10.18 million (95% UI = 4.30; 16.29).

### 3.3 Global trends in CKD-T2DM burden attributable to dietary risk factors across regions

**Figure 6:**
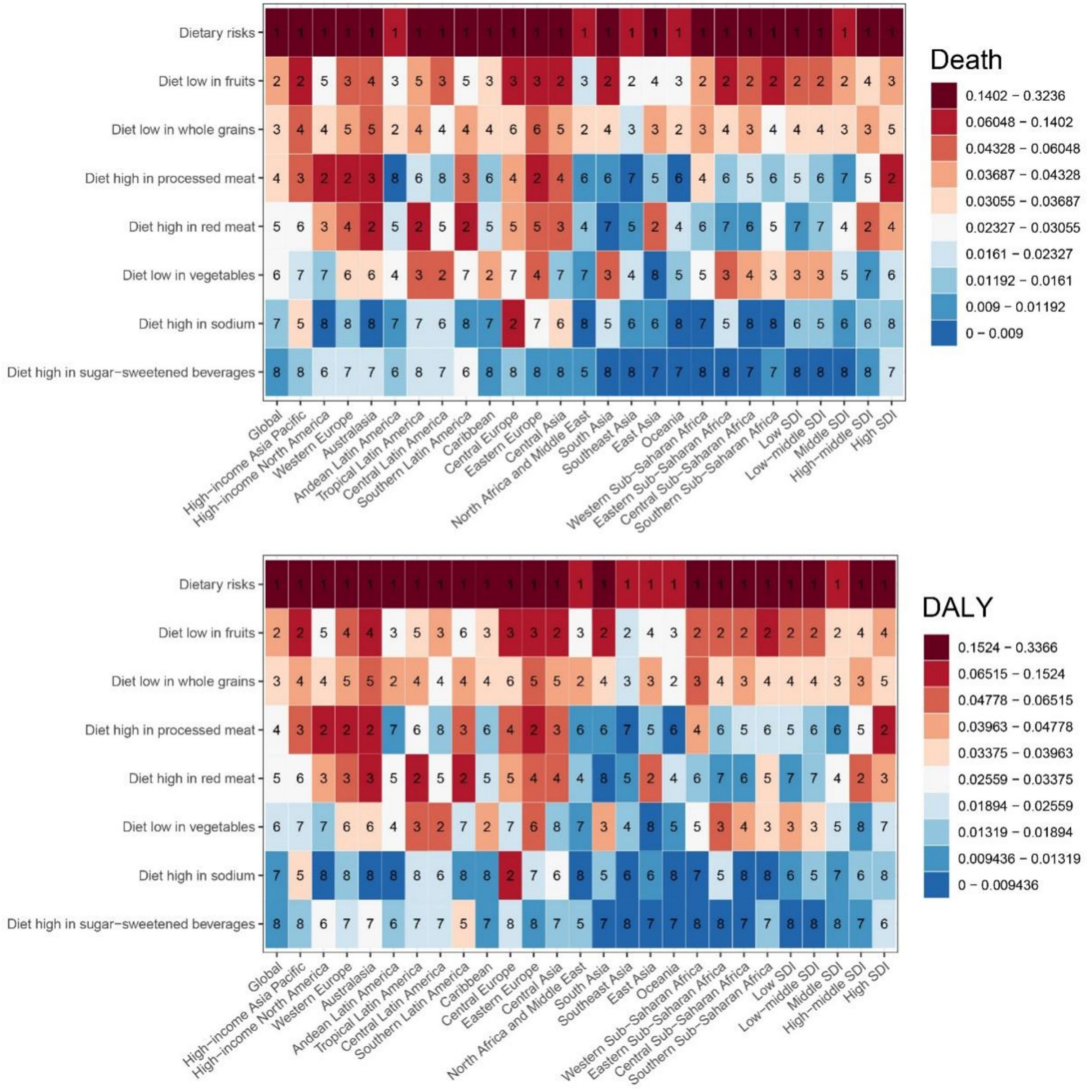
The proportion of deaths and DALYs for CDK-T2DM attributable to specific dietary risk factors in 2021.

Figure 6 illustrates the proportions of CKD-T2DM attributable to individual dietary risks for ASMR and ASDR in 2021 across 21 GBD and 5 SDI regions globally. The dietary risk factor “high intake of processed meats” shows higher proportions in high-income North America, Western Europe, Australasia, Eastern Europe, Southern Latin America, high-income Asia Pacific, Central Europe, and Central Asia. Similarly, “high intake of red meat” follows a comparable pattern, with the highest proportions in Australasia, Andean Latin America, and Southern Latin America, followed by Central Asia, Western Europe, East Asia, Central Europe, and high-income North America. The highest percentages of ASMR and ASDR attributable to “low fruit intake” are found in Eastern Europe, Central Asia, South Asia, High-income Asia Pacific, Central Europe, Southern Sub-Saharan Africa, and Eastern Sub-Saharan Africa. While Australasia, High-income Asia Pacific, and Eastern Europe showed the highest ASMR and ASDR percentages associated with “low whole grain intake.”

In high SDI regions, a clear trend of higher percents for diets rich in processed meats, low in fruits, and high in red meat was observed. Specifically, in high SDI regions, the value for “high processed meat intake” is 8.3%, “diet low in fruits” is 4.2%, and “high red meat intake” is 3.8%. In mid-high SDI regions, “high red meat intake” and “diet low in whole grains” have higher values of 4.3 and 4.1%, respectively. Conversely, in low and mid-low SDI regions, dietary patterns shift noticeably, with higher values for low fruit and vegetable intake. For instance, in low SDI regions, the value for “low fruit intake” is 6.0%, and “low vegetable intake” is 4.3%, while in mid-low SDI regions, “low fruit intake” is 6.0% and “low vegetable intake” is 3.9%. Figure 6 indicates that as the SDI increases, dietary patterns shift from low fruit and vegetable intake to higher intake of processed meats and red meats, with a concurrent reduction in whole grain consumption.

### 3.4 The impact of SDI on the burden of CKD-T2DM attributable to dietary risk factors

**Figure 7:**
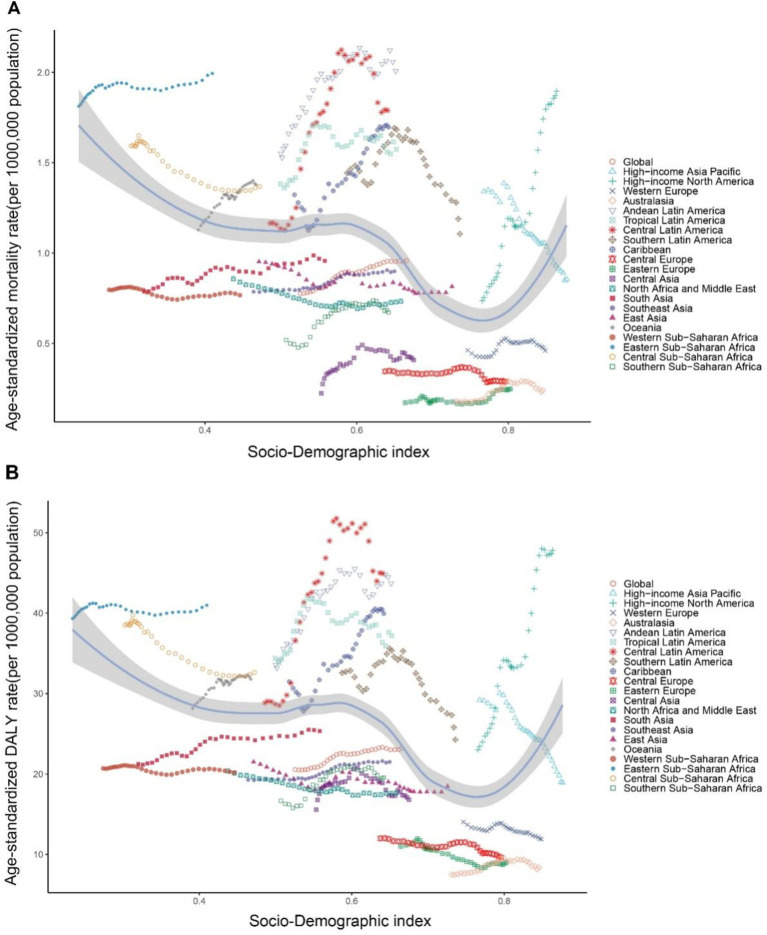
Age-standardized rates of CDK-T2DM attributable to dietary factors across 21 Global Burden of Disease regions by Socio-Demographic Index, 1990–2021. **(A)** Mortality. **(B)** DALYs.

As SDI values increase, there is heterogeneity in the mortality and DALY rates of CKD-T2DM attributable to dietary risk factors across regions (Figure 7). From 1990 to 2021, despite fluctuations, the overall CKD-T2DM mortality and DALY rates attributable to dietary risk factors generally increased with higher SDI values in Eastern Sub-Saharan Africa, Andean Latin America, Central Latin America, Tropical Latin America, the Caribbean, Southern Sub-Saharan Africa, Central Asia, South Asia, Eastern Europe, Oceania, Australasia, and High-Income North America, with the fastest increase observed in High-Income North America. Conversely, Central Sub-Saharan Africa, Western Sub-Saharan Africa, North Africa and the Middle East, East Asia, Southern Latin America, Central Europe, Western Europe, and High-Income Asia Pacific show a significant decrease in mortality and DALY rates attributable to dietary risk factors with increasing SDI. Notably, Eastern Sub-Saharan Africa has higher CKD-T2DM DALY and mortality rates, which are markedly higher than those in other regions with the same SDI value (Figure 7). Our correlation analysis shows that ASMR is significantly negatively correlated with SDI (r = −0.368, *p <* 0.001). Similarly, the ASDR model shows a significant negative correlation with SDI (r = −0.385, *p <* 0.001).

**Figure 8:**
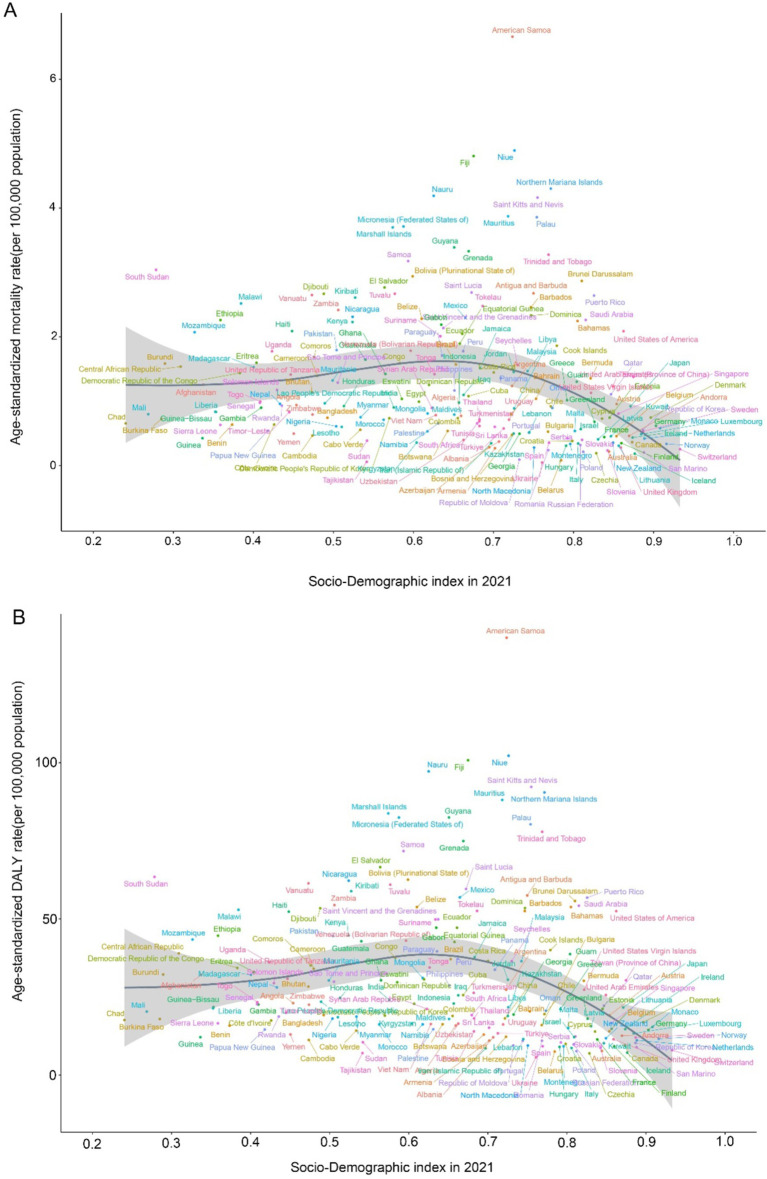
Age-standardized rates of CDK-T2DM attributable to dietary factors across 204 countries and territories by Socio-Demographic Index in 2021. **(A)** Mortality, **(B)** DALYs.

In our correlation analysis of 204 countries, ASMR shows a significant negative correlation with SDI (r = −0.342, *p <* 0.001). Similarly, ASDR also exhibits a significant negative correlation with SDI (r = −0.3608, *p <* 0.001) (Figure 8).

### 3.5 Age-standardized mortality and DALY rate trends predicted by Bayesian age-period-cohort (BAPC)

**Figure 9:**
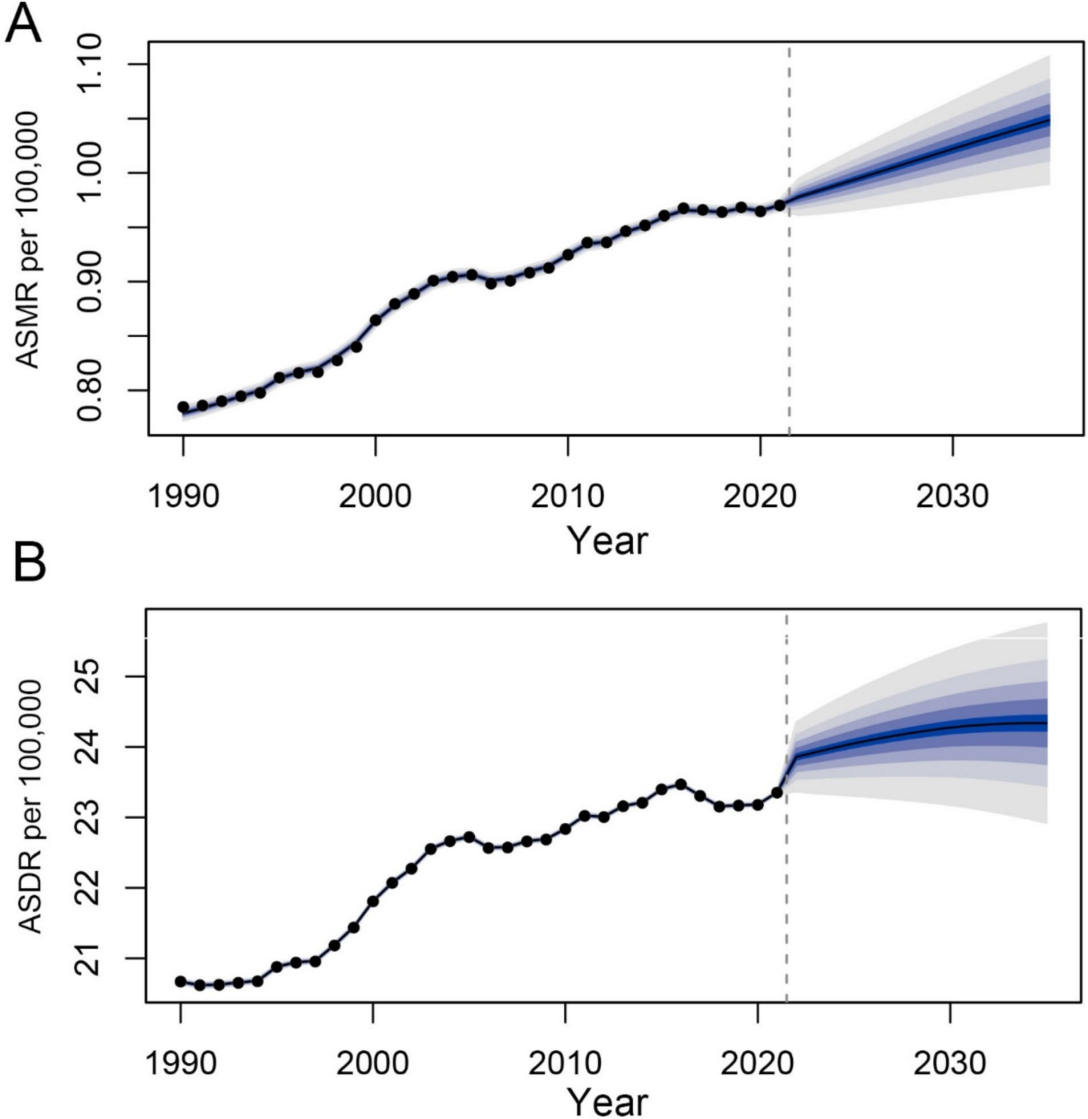
Prediction of the death and DALY rates of CDK-T2DM attributable to dietary factors from 2021 to 2035 in age-standardized population **(A)** the age-standardized death rate (ASDR) of CDK-T2DM. **(B)** The age-standardized DALY rate of CDK-T2DM.

To forecast the trends in age-standardized mortality and DALY rates for 2035, we performed BAPC analysis. The results are summarized in Figure 9. Overall, the predictions indicate that age-standardized mortality and DALY rates are expected to rise in the coming years. Specifically, the global age-standardized mortality rate is projected to be approximately 1.05, and the DALY rate is anticipated to be around 24.33 by 2035.

## 4 Discussion

The increasing mortality and DALY rates associated with CKD-T2DM can be attributed to several factors (20, 21). Population growth and aging are significant drivers of the CKD-T2D burden. Additional contributors include high fasting blood glucose levels, high systolic blood pressure, behavioral factors, dietary factors, environmental/occupational risks, and high body mass index, all of which exacerbate the burden of type 2 diabetes kidney disease (22, 23).

Our analysis of the GBD data provides a comprehensive overview of the global burden of type 2 diabetes kidney disease attributed to dietary risks from 1990 to 2021. The study highlights a significant increase in mortality and DALY rates over the past 32 years, with dietary risk factors—such as low fruit intake and high consumption of red and processed meat—making substantial contributions. Notably, while the contribution of diet in high SSB is not among the top three, its rate of increase is the most rapid. Research indicates that processed red meat, contains high levels of saturated fat, heme iron, sodium, nitrate and additives that can lead to insulin resistance, oxidative stress and inflammation (24). SSBs also adversely affect blood glucose (7). Conversely, certain fruits are rich in fiber, vitamins, minerals, antioxidants, and polyphenols, which can help regulate blood glucose levels and improve insulin sensitivity (25–27). Whole grains can reduce postprandial blood glucose and peripheral insulin resistance (28). As the SDI rises, dietary patterns shift from a lack of fruits and vegetables to a diet rich in processed and red meat but low in whole grains The so-called Western lifestyle is closely linked to the rapid pace of global urbanization and technological advancement. This suggests that addressing dietary quality through public health, clinical, and policy measures could help mitigate the global burden of CKD-T2D mortality and DALY. However, the PAF of dietary risks in 2021 was lower than in 1990. This may be attributed to the significant increase in the use of medications during the COVID-19 pandemic, along with the surge in stress, anxiety, depression, and other mental health conditions associated with drug use. These factors likely contributed to a portion of the deaths and DALYs resulting from other outcomes (15).

Furthermore, the data indicate a negative correlation between SDI values and the burden of CKD-T2D due to dietary risk factors. This may be because higher SDI countries have more financial resources to establish better healthcare systems, leading to more effective and timely treatment for type 2 diabetes kidney disease (29). This is also a positive finding, suggesting that progress has been made in the treatment of CKD resulting from type 2 diabetes (30).

The distribution of deaths attributable to dietary risk factors for type 2 diabetes kidney disease by sex and age in 2021 shows a higher burden among the elderly, peaking in women and men aged 70–74. Both female and male deaths from CKD-T2D peak in the elderly, which aligns with global and regional trends indicating that mortality and incidence rates increase with age due to the higher occurrence of microvascular complications with long-term diabetes (31, 32). Our results reveal that among men under 80–84 years, deaths attributable to dietary risk factors for CKD-T2DM exceed those in women; above this age, women surpass men. Additionally, men under 74 experience higher DALY from dietary risk factors for CKD-T2DM compared to women, while women older than 75–79 years have higher DALY than men. These findings may be explained by biological and socio-psychological factors, such as the protective role of estrogen, which declines with age, and related social factors, including a higher likelihood of women seeking screening or diagnosis compared to men (33, 34).

From 1990 to 2021, the geographical heterogeneity in the trends of CKD-T2DM attributable to dietary factors has been substantial. Regions such as High-income North America and Central Latin America have experienced the greatest annual increases in ASMR and ASDR. While the overall burden in regions such as High-income Asia Pacific and Central Sub-Saharan Africa have significantly decreased, global trends show an increase, with forecasting models indicating a rising burden. The BAPC (Age-Period-Cohort) model, which has been validated as a predictive model, was used to analyze and forecast trends in age-standardized mortality and DALY rates (19, 35). Our findings suggest that the age-standardized mortality and DALY rates attributable to dietary risks for CKD-T2DM are projected to rise in the coming years. Thus, early identification of high-risk individuals is helpful to timely intervene and reduce the risk of T2D and its complication (36–38). However, it is important to interpret this prediction with caution, as the increasing life expectancy and aging population may exacerbate the situation. Additionally, we observed that in 2021, within the same SDI context, the mortality and DALY rates for CKD-T2DM of certain countries exhibited exemplary performance, indicating significant opportunities to narrow the gap. This should encourage countries with similar SDI to optimize their available resources for better health outcomes. Therefore, appropriate dietary management for CKD-T2DM could hold substantial value (39).

This study has several limitations. Firstly, we rely on estimates generated by the GBD study group. Although GBD methods and results are considered reliable and robust, they are inevitably constrained by the quality of available data. Secondly, the modeling methods used available dietary risk data to estimate the risk but do not establish causal relationship and should be viewed as approximations of risk. Additionally, data insufficiencies in many parts of the world can lead to inaccuracies in the results, particularly in resource-limited regions. To mitigate the impact of data scarcity, we focused on regional rather than individual country data. Caution is advised when comparing data across countries due to inconsistencies in data sources and differences in risk factors among countries and income levels, which also pose challenges to accurately estimating the burden (40). Our analysis further categorized the data by variables such as age, gender, SDI, and geographic region; however, the lack of comprehensive and reliable data on other social determinants of health limits our understanding of global differences related to dietary factors and CKD-T2DM. Moreover, the study does not include other dietary factors that might impact CKD-T2DM, potentially leading to an underestimation of the burden caused by dietary factors. Given that disease burden is typically calculated based on current exposure and health outcomes, discrepancies may arise due to the differing time dimensions of risk and outcomes (15). Additionally, the calculation of PAF typically assumes that risk factors operate independently, whereas in reality, they may interact synergistically or antagonistically, complicating the disease burden attributable to each risk factor. Finally, the SDI used in GBD 2021 is a regional-level indicator and does not account for potential patient-level confounding factors, limiting its applicability for individual risk assessment.

From a public health perspective, the increasing global mortality and disability rates due to dietary risk factors for CKD-T2DM over the past 32 years necessitate urgent intervention. Dietary screening and education can improve the quality of life for high-risk populations and reduce disease burden (41). Public health campaigns targeting high-risk groups, raising awareness of the link between CKD-T2DM and high red or high processed meat consumption or low fruit and grain intake, promoting balanced diets, and regulating the availability of high-risk foods can play a crucial role in curbing the epidemic of CKD-T2DM (42). Imposing tax on sugary drinks and integrating dietary risk factors into national health surveillance programs could be an effective strategy to reduce the associated burden (43, 44).

## 5 Conclusion

The increasing global burden of CKD-T2DM attributable to dietary risks underscores the urgent need for public health interventions focused on diet quality. This study employs 2021 Global GBD data to conduct a multidimensional and longitudinal analysis of CKD-T2DM across various regions and countries, attributed to seven dietary risks. By identifying the most influential dietary risk factors (low in fruits, high in red and high in processed meat), the most rapidly increasing factor (diet high in sugar-sweetened beverages) and predicting their trend, our research provides actionable insights that could serve as a foundation for future research and policy aimed at mitigating the global burden of CKD-T2DM.

## Data Availability

The original contributions presented in the study are included in the article/supplementary material, further inquiries can be directed to the corresponding authors.

## Footnotes

^1^ https://vizhub.healthdata.org/gbd-results/
